# Anti-inflammatory and antibacterial activities of cerium-containing mesoporous bioactive glass nanoparticles for drug-free biomedical applications

**DOI:** 10.1016/j.mtbio.2021.100150

**Published:** 2021-10-14

**Authors:** F. Kurtuldu, H. Kaňková, A.M. Beltrán, L. Liverani, D. Galusek, A.R. Boccaccini

**Affiliations:** a FunGlass, Alexander Dubček University of Trenčín, 911 50 Trenčín, Slovakia; b Institute of Biomaterials, Department of Materials Science and Engineering, University of Erlangen-Nuremberg, 91058 Erlangen, Germany; c Departamento de Ingeniería y Ciencia de Los Materiales y Del Transporte, Escuela Politécnica Superior, University of Seville, 41011 Seville, Spain; d Joint Glass Centre of the IIC SAS, TnUAD and FChFT STU, FunGlass, 911 50, Trenčín, Slovakia

**Keywords:** Metal ion, Bioactive glassses, Nanoparticles, Anti-inflammation activity, Antibacterial materials

## Abstract

Mesoporous bioactive glass nanoparticles (MBGNPs) are attracting significant attention as suitable materials for multifunctional biomedical applications. In this study, cerium was incorporated into MBGNPs using two different approaches. In the first approach, cerium was added to the glass system directly during the synthesis, while in the second approach, cerium was added to the as-synthesized MBGNPs via the template ion-exchange method. The influence of the method of synthesis on the physicochemical properties of nanoparticles was examined by SEM, TEM, XRD, FTIR, and N_2_ adsorption-desorption analyses. The MBGNPs exhibited spheroidal morphology and disordered mesoporous structure. XRD analysis confirmed the amorphous nature of the nanoparticles. The chemical composition was determined by the acid digestion method using ICP-OES. The influence of the synthesis method on the specific surface area, mesoporosity, and solubility of synthesized nanoparticles in Tris/HCl (pH 7.4) and acetate (pH 4.5) buffer has also been studied. The obtained Ce containing MBGNPs were non-cytotoxic toward ​preosteoblast MC3T3-E1 cells in contact with nanoparticles in a concentration of up to 100 ​μg/mL. The anti-inflammatory effect of Ce containing MBGNPs was tested with lipopolysaccharides (LPS)-induced proinflammatory RAW 264.7 macrophage cells. Ce containing MBGNPs decreased the release of nitric oxide, indicating the anti-inflammatory response of macrophage cells. Ce containing MBGNPs also showed antibacterial activity against *S. aureus* and *E. coli*. The mentioned features of the obtained MBGNPs make them useful in a variety of biomedical applications, considering their biocompatibility, anti-inflammatory response, and enhanced antibacterial effect.

## Graphical abstract

**Figure:**
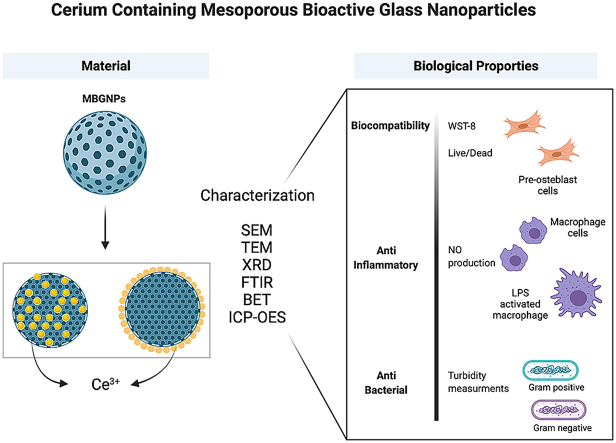

## 1 Introduction

Bioactive glasses (BGs) are an extraordinary class of biomaterials suitable for numerous biomedical applications, from bone regeneration and wound healing to cancer treatment [[1], [2], [3]]. BGs are osteoconductive, osteoinductive ​and have an excellent mineralization capability [4]. They can form strong interfacial bonding with both hard and soft tissues [4]. Furthermore, depending on their composition and morphology, BGs could enhance vascularization, wound healing, anti-inflammatory responses, and inhibit bacteria growth [5]. Recently, mesoporous bioactive glass nanoparticles (MBGNPs) have drawn attention among the various types of BGs [[6], [7], [8], [9]]. MBGNPs have been used as drug delivery carriers, bioactive fillers in composite biomaterials, or injectable biomaterials due to their large specific surface area, high pore volume, uniform pore size, high drug loading capacity, and flow properties [9,10]. MBGNPs are mainly composed of silica networks incorporating calcium and are characterized by a mesoporous structure [11]. Additional therapeutic ions can be incorporated into the silica network, such as Cu, Zn, Mg, Ag, Ga, and Ce, in order to provide specific biological functions (e.g. ​osteogenesis, angiogenesis, anti-inflammatory response, antibacterial activity) to MBGNPs [10,12]. In this way, MBGNPs can be tailored to perform dual delivery of drugs (e.g. ​antibiotics, enzymes, growth factors) and biologically active ions ​for specific biomedical applications [13]. Thus, MBGNPs have acquired increasing attention in nanomedicine, cancer therapy, tissue regeneration, and wound healing applications.

Biomaterials are also developed with the aim of controlling the levels of reactive oxygen species (ROS) [14], which are a product of cellular oxidative metabolism [15]. They play a significant role in cell survival, death, differentiation, and the production of inflammation-related factors [15,16]. For example, in normal physiological conditions, ROS have an important role in osteoclastogenesis, and they help the bone renewal process by promoting the resorption of bone tissue [17]. Despite these beneficial activities, excessive ROS can be toxic to cells [18]. The removal of ROS is normally achieved through various detoxification mechanisms like enzymatic reactions (such as superoxide dismutase) and the action of non-enzymatic molecules like antioxidants [15,16,19]. ROS production and antioxidant defenses should be in balance; otherwise, oxidative stresses occur [15]. Oxidative stresses cause a series of deregulations of cellular functions, which can lead to damage of DNA, proteins, and cells ​and induce inflammatory responses [15,18]. BGs containing biologically active ions (e.g. ​cerium, selenium) could be used to control the ROS level, which further promotes desired biological activities like osteogenesis ​or anti-inflammatory response [[18], [19], [20]].

Inorganic materials like BGs have been widely applied in tissue regeneration applications [1,2,21]. Existing research recognizes the critical role played by therapeutic metallic ion addition to BGs for osteogenesis and angiogenesis [10]. Moreover, BG nanoparticles containing therapeutic ions, such as cerium, can exhibit extra effects on inflammatory response and antibacterial activity. Cerium is a therapeutic ion with unique biological activity. Its ability to change the oxidative state between Ce^3+^ and Ce^4+^ during redox reactions is a distinctive feature that gives Ce a dual role in biological systems [22,23]. In physiological pH conditions, it can act as an antioxidant (Ce^3+^) and ROS scavenger, mediating the oxygen within the microenvironment, and as a result, cerium induces anti-inflammatory response and osteogenesis [22,24]. On the other hand, cerium takes the role of pro-oxidant (Ce^4+^) under low pH conditions, characteristic of bacterial infections or tumorous tissue environments [25]. Thus, Ce generates ROS and causes cell damage, showing antibacterial activity [25].

A considerable amount of literature has been published on cerium-containing BGs [20,[26], [27], [28], [29], [30], [31]]. For example, Shruti et al. [29] studied cerium-containing mesoporous bioactive glass (MBGs, (80-X)SiO_2_–15CaO–5P_2_O_5_-XCe_2_O_3_ in mol%) containing up to 1 ​mol% Ce_2_O_3_. The study showed that cerium did not affect the mineralization capacity and textural properties of MBGs. Varini et al. [32] studied cerium-containing MBGs and alginate composite biomaterials. They showed that cerium addition improved preosteoblast cell proliferation and reduced oxidative stress. Antibacterial properties of cerium-containing MBGs have also been reported in the literature [33]. The addition of 5 and 10 ​mol% of cerium into the MBGs (50SiO_2_-(45-X)CaO–5P_2_O_5_–XCeO_2_ in mol%) was shown to inhibit *E. coli* bacteria strain. Cerium in oxide form (especially nanoceria) is also used in biomedical applications. Pinna et al. [23] recently incorporated nanoceria into mesoporous silica nanoparticles (MSNs) to stimulate bone regeneration and reduce osteoclast activity. The study showed that nanoceria addition improved the osteogenic properties of MSNs and showed antioxidant properties. Moreover, El-Fiqi et al. [30] prepared bioactive glass nanoparticles (SiO_2_-(15-X)CaO-xCe_2_O_3_) containing up to 10 ​mol% Ce_2_O_3_ with size less than 30 ​nm, and the nanoparticles showed good antioxidant properties. Additionally, cerium-containing MBGNPs in the SiO_2_–CaO system were synthesized with a two-step approach, using the postmodification method [20,34]. The Ce-containing MBGNPs exhibited anti-inflammatory effects and pro-osteogenic activity [20]. The synthesis of cerium-containing MBGNPs in the system of SiO_2_–CaO with a one-step approach by microemulsion assisted sol-gel method was reported in our recent publication [35]. The study confirmed the bioactive behavior of the prepared MBGNPS doped with 1 ​mol% Ce_2_O_3_. Additionally, the elution extract of MBGNPs showed antibacterial behavior and significantly increased the viability of MG-63 osteoblast-like cells. However, the effect of different incorporation methods of cerium into MBGNPs has not yet been investigated in depth. There are two different methods commonly used to add metal ions into MBGNPs, namely direct addition during synthesis and postmodification methods [10]. In this study, we used both approaches. First, cerium was added directly during the sol-gel synthesis of MBGNPs in the SiO_2_–CaO system, and second, the postmodification method was used to add cerium. Both methods showed differences in NPs morphology, ion release profile, and protein adsorption capabilities.

The aim of this study was thus to synthesize cerium-containing MBGNPs for multifunctional biomedical application with anti-inflammatory response and antibacterial activity. We report the synthesis of highly dispersed cerium-containing MBGNPs by using both microemulsion-assisted sol-gel method and template ion-exchange method (TIE). The physicochemical and structural properties of cerium-containing MBGNPs were comprehensively investigated using SEM, TEM, FTIR, XRD, N_2_ adsorption-desorption, and ICP-OES technique. The influence of Ce addition on protein adsorption, biocompatibility, anti-inflammatory response, and antibacterial activity was evaluated and discussed.

## 2 Materials and method

### 2.1 Materials

Tetraethyl orthosilicate (TEOS, (C_2_H_5_O)_4_Si 99%, Sigma-Aldrich), cetyltrimethylammonium bromide (CTAB, CH_3_(CH_2_)_15_N(Br)(CH_3_)_3_, BioXtra, ≥99%, Sigma-Aldrich), ethyl acetate (EA, CH_3_COOC_2_H_5_, Merck, Germany), ammonium hydroxide (NH_4_OH ACS reagent, 28% NH_3_ basis, Sigma-Aldrich), calcium nitrate tetrahydrate (Ca(NO_3_)_2_.4H_2_O, 99.1%, VWR) and cerium nitrate hexahydrate (CN, Ce(NO_3_)_3_.6H_2_O, 99%, Sigma-Aldrich, Germany) were used for the synthesis. All chemicals were of analytical grade.

### 2.2 Synthesis of Ce containing MBGNPs

Cerium-containing MBGNPs in the SiO_2_–CaO binary system were produced with two different approaches. In the first one, cerium was added to the MBGNPs directly during the synthesis, and in the second approach, cerium was incorporated into the MBGNPs via the template ion exchange (TIE) method.

MBGNPs were synthesized using a microemulsion-assisted sol-gel method. These nanoparticles were prepared by adapting the procedure used by Liang et al. [36]. The procedure is described briefly in this section. First, 2.8 ​g of CTAB were mixed with 150 ​mL of Milli-Q water at 30 ​°C, until it was completely dissolved. Then 40 ​mL of EA were added, and the solution was left stirring for 30 ​min. Next, 3.66 ​mL ammonium hydroxide was added, and the solution was left stirring for 15 ​min. Afterward, 14.4 ​mL of TEOS and appropriate amounts of calcium nitrate tetrahydrate and cerium nitrate hexahydrate were added stepwise in 30 ​min ​intervals, and the solution was left stirring for 4 ​h. Then, the precipitates were separated from the solution via centrifugation and washed twice with Milli-Q water and one time with ethanol. Before the final calcination step, the collected nanoparticles were dried at 60 ​°C overnight. Finally, the dried samples were heated at 1 ​°C/min to 650 ​°C with 3 ​h of holding time. The sample containing only calcium was labeled as Ca1 (nominal composition 70SiO_2_–30CaO (mol%)). The sample containing calcium and cerium was labeled as Ce1 (nominal composition 70SiO_2_–29CaO–1Ce_2_O_3_ (mol%)).

**Fig. 1:**
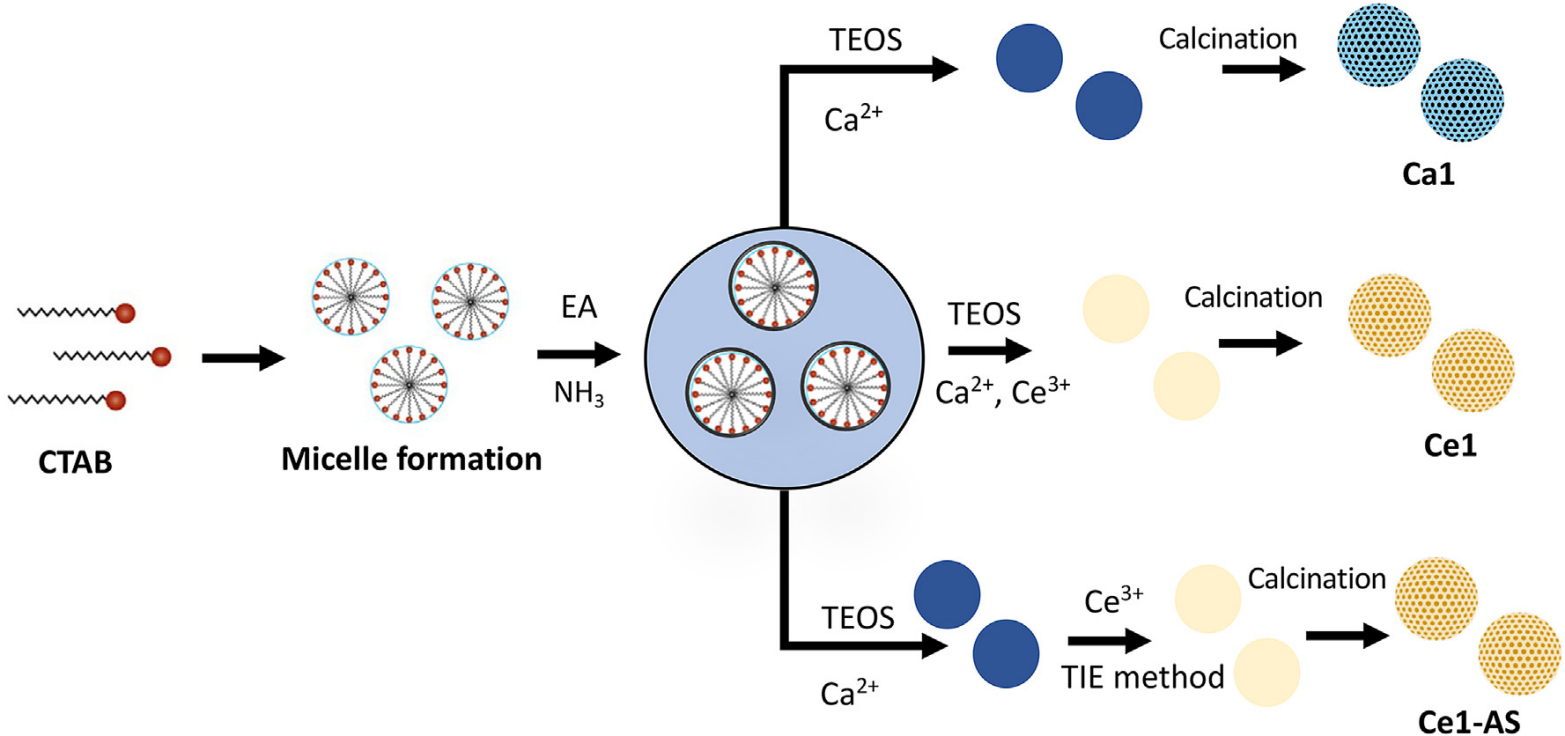
Schematic illustration of MBGNPs synthesis and the TIE method.

In the second approach, cerium was incorporated in the MBGNPs by the template ion exchange method [37]. The as-synthesized Ca1 (2.00 ​g, without calcination) was stirred in 50 ​mL of the aqueous solution of 1·10^−2^ ​mol/L cerium nitrate hexahydrate at ambient temperature for 1 ​h. Then, the mixture was kept in an oil bath at 80 ​°C for 20 ​h without stirring. Afterward, the same washing and calcination procedures were applied to the samples as described above. The samples synthesized via the template ion-exchange method are denoted as Ce1-AS. Fig. 1 shows the schematic illustration of the MBGNPs synthesis and the TIE method.

### 2.3 Physicochemical characterization of MBGNPs

The morphology and surface microstructures of the nanoparticles were characterized using a scanning electron microscope (SEM, Auriga, Zeiss, Germany) ​and a (scanning) transmission electron microscope ((S)TEM, FEI Talos F200S, Netherlands) equipped with Super-X energy dispersive X-ray spectrometry (EDX) system, including two silicon drift detectors. For SEM examination, the samples were dispersed in EtOH by ultrasonication, a drop was placed on an aluminum holder and dried. SEM images were taken at accelerating voltages between 1 ​kV and 3 ​kV. For TEM observation, the samples were dispersed in EtOH by ultrasonication and were deposited on a Holey Carbon Film on a copper grid. The (S)TEM microscope was operated at an accelerating voltage of 200 ​kV. The average particle size of the nanoparticles was determined via ImageJ software (version 1.53) using SEM images. For each sample, a minimum of 100 particles was measured.

X-ray powder diffraction (XRPD) analysis was performed using an X-ray diffractometer (Empyrean, Malvern Panalytical, Almelo, Netherlands) using Cu-Kα radiation (45 ​kV) in the 2Θ range 2°–80°.

Fourier transform infrared spectroscopy (FTIR) analyses were performed on the samples using an IRAffinity-1S spectrophotometer (SHIMADZU, Japan) in absorbance mode in the wavenumber range from 4000 to 400 ​cm^−1^ ​at a resolution of 4 ​cm^−1^.

The Brunauer-Emmett-Teller (BET) surface area and the Barrett-Joyner-Halenda (BJH) pore size distribution of MBGNPs were determined using nitrogen adsorption-desorption analysis on ASAP 2020 (Micromeretics, USA) with isotherm at 77 ​K.

The quantitative chemical composition of MBGNPs was determined using an inductively coupled plasma-optical emission spectrometry (ICP-OES, Agilent 5100 SVDV) via the acid digestion method. For dissolving the MBGNPs for the analysis, the microwave-assisted digestion (Speedwave 4, Berghof Products ​+ ​Instruments, Germany) was applied using a digestion solution prepared from 6 ​mL of HCl (ACS 37%, Sigma-Aldrich), 2 ​mL of HNO_3_ (65%, Centralchem, Slovakia), and 0.5 ​mL of HF (38–40%, Penta s.r.o., Czech Republic). Afterward, 5 ​mL of H_3_BO_3_ (99.5%, Centralchem, Slovakia) was added to the acid mixture to avoid corrosion of silica parts of the instrument by hydrofluoric acid and to prevent the formation of poorly soluble fluorides in the presence of alkali earth elements [38]. As internal standards, the multielement solution was prepared by adding Sc (10 ​mg/L) and Be (1 ​mg/L). Since silica forms volatile complexes (like H_2_SiF_6_) in the presence of HF, its content could not be measured and was calculated by adding to 100%.

### 2.4 Ion release behavior of MBGNPs

The ion release test was performed in Tris/HCl (Tris base, Sigma-Aldrich/ACS 37%, Sigma-Aldrich) buffer (50 ​mM, pH 7.4) and sodium acetate/acetic acid (99%, Sigma-Aldrich/99%, Penta s.r.o., Czech Republic) buffer (0.1 ​M, pH 4.5). 1.5 ​mg/mL of MBGNPs were incubated in the medium at 37 ​°C with 120 ​rpm agitation speed up to 3 days. After immersion, elemental analysis was carried out using the ICP-OES (Agilent 5100 SVDV). Each sample was analyzed in three replicates.

### 2.5 Protein adsorption

The protein adsorption ability of MBGNPs was determined with the Pierce™ BCA protein assay kit (Thermo Fisher, Germany), according to the manufactures' instruction. The nanoparticles were soaked in 1 ​mg/mL bovine serum albumin (BSA; Sigma-Aldrich) solution in Milli-Q water for 2 and 4 ​h at 37 ​°C. At the determined time points, 25 ​μL supernatants were collected and added to a 96-well plate. Then 200 ​μL working reagent was added to each well, and the plate was mixed thoroughly with a plate shaker for 1 ​min. Then the plate was incubated at 37 ​°C for 30 ​min. The absorbance was measured with a microplate reader (FLUOstar Omega, BMG Labtech, Germany) at 562 ​nm.

### 2.6 Cell culture

The preosteoblast MC3T3-E1 and murine macrophage RAW 264.7 cell lines were used in the study. MC3T3-E1 cells were cultured in α-MEM (Gibco, Germany) supplemented with 10% Fetal Bovine Serum (FBS, Corning, USA), 1% l-Glutamine (Gibco, Germany), and 1% Penicillin-Streptomycin (PS, Gibco, Germany) at 37 ​°C in a humidified incubator with 5% CO_2_. Cells were harvested using trypsin-EDTA (0.25%, Gibco, Germany) and resuspended in the cell culture medium. RAW 264.7 ​cells were cultured in a low glucose DMEM medium (Gibco, Germany) supplemented with 10% FBS and 1% PS at 37 ​°C in a humidified incubator with 5% CO_2_. Cells were harvested using a cell scraper and resuspended in the cell culture medium. In this study, direct in vitro cytotoxicity test and cell staining were performed using MC3T3-E1 cells, and an anti-inflammatory assay was performed by using RAW 264.7 ​cells.

### 2.7 In vitro cytotoxicity

MC3T3-E1 cells viability, after direct exposure to MBGNPs over time, was evaluated using WST-8 assay (CCK-8 Kit, Sigma-Aldrich, Germany). In each well of 48-well plates, 10^5^ ​cells/mL were seeded and allowed to grow for 24 ​h. Afterward, the cells were directly exposed to Ca1, Ce1, and Ce1-AS at a concentration of 5, 10, 50, 100, and 500 ​μg/mL. Cells cultured in cell culture medium without MBGNPs were used as a positive control. Cells cultured in the culture medium with 6 ​vol% dimethyl sulfoxides (DMSO) were used as a negative control. Cell viability was measured after 48 ​h of direct contact with MBGNPs. Then, the cell culture medium was removed from each well, and the cells were rinsed three times with phosphate buffer saline (PBS, Gibco, Germany) in order to remove all MBGNPs. Afterward, 1% v/v WST-8 in a cell medium solution was added to each well and further incubated for 3 ​h. The solutions were transferred into a 96-well plate to measure absorbance at 450 ​nm using an Elisa microplate reader (FLUOstar Omega, BMG Labtech, Germany). All experiments were made in triplicate. The viability of the MC3T3-E1 cells was calculated according to the following equation:

$$Cell\;viability\;\left(\%\right)=\;\frac{\left(Absorbance\;of\;sample-Absorbance\;of\;blank\right)}{\left(Absorbance\;of\;positive\;control-Absorbance\;of\;blank\right)}\times 100$$

For investigating cell viability further, the cells were stained with calcein acetoxymethyl-ester (Calcein-AM, Thermo Fisher Scientific, Germany) and propidium iodide (PI, Thermo Fisher Scientific, Germany), which selectively stain live or dead cells. Cells were washed with PBS to remove the cell culture medium and nanoparticles. Afterward, 4 ​μg/mL calcein-AM and 5 ​μg/mL propidium iodide containing Hank's balanced salt solution (HBSS, Gibco, Germany) were added to the cells and incubated for 30 ​min in the dark. Then the cells were put in a fixing solution (4% (w/v) paraformaldehyde in PBS) for 15 ​min. Fluorescence images were taken using a fluorescence microscope (Axio observer, Carl Zeiss).

### 2.8 Fluorescence microscopy assays

For evaluating cell morphology after 48 ​h in contact with the MBGNPs, MC3T3-E1 cells were cultured in 48-well plates at a density of 10^5^ ​cells/mL in the presence of 5, 10, 50, 100, and 500 ​μg/mL nanoparticles. Then the cells were rinsed three times with PBS to remove all nanoparticles. The cells were fixed in 4% (w/v) paraformaldehyde in PBS for 15 ​min and permeabilized with Triton-X 100 containing permeabilization buffer for 5 ​min. F-actin staining was then performed with rhodamine phalloidin (R415, molecular probes, Thermo Fisher Scientific, Germany). The cell nuclei were visualized with blue ​fluorescent stain, DAPI (4′,6-Diamidino-2-Phenylindole, Dihydrochloride, Thermo Fisher Scientific, Germany). Afterward, the cells were washed three times with PBS, and images were taken by fluorescence microscopy (Axio observer, Carl Zeiss).

### 2.9 Anti-inflammatory study

RAW 264.7 ​cell line was used for determining the anti-inflammatory activity of MBGNPs. For evaluating the anti-inflammatory effect of MBGNPs, the cells were treated with lipopolysaccharides (LPS, Sigma) to start the inflammation. Briefly, around 10^5^ ​cells/mL were seeded in each well of 48-well plates and were left to attach for 24 ​h. After that 1 ​μg/mL LPS-containing cell culture medium (DMEM) with 1% FBS was added and kept in contact with the cells for 12 ​h. Then, the cell culture media were exchanged by normal cell culture media with the addition of Ca1, Ce1, and Ce1-AS at a concentration of 50 ​μg/mL. After 24 ​h, the cell culture medium was taken, and nitric oxide (NO) amount was determined using Griess reagent kit (G7921, Thermo Fisher, Germany), according to the manufacturer's instruction.

### 2.10 Antibacterial activity

The antibacterial properties of MBGNPs against *E. coli* and *S. aureus* were studied. Both bacteria strains were cultivated in lysogeny broth (LB) medium (Luria/Miller) at 37 ​°C. The antibacterial activity of MBGNPs was assessed quantitatively based on the absence of light caused by the turbidity of the bacterial suspension. The samples were added in LB medium at the concentrations of 10, 1, 0.5, 0.1, 0.05 and 0.01 ​mg/mL. Then the 96-well plate was inoculated with 150 ​μL of nanoparticle/LB medium suspension and 50 ​μL of bacterial suspension (1–5 × 10^5^ ​CFU/mL) at 37 ​°C for 24 ​h. The samples without MBGNPs were used as a positive control. Optical density (OD) values at 600 ​nm were measured via a microplate reader (PHOmo, Anthos Mikrosysteme GmbH, Germany). By considering the possible interference from the nanoparticles, the OD values of the samples without bacteria addition were substituted as the background with each glass and corresponding concentration. All experiments were made in triplicate. The viability of the bacteria was calculated according to the following equation:

$$Relative\;bacteria\;viability\;\left(\%\right)=\;\frac{OD_{Sample}}{OD_{Control}}\times 100$$

### 2.11 Statistical analysis

Statistical analysis was performed using one-way ANOVA and Bonferroni's test. The statistical tests were conducted with the help of Origin software (OriginLab 2017, USA). The results were expressed as mean ​± ​standard deviation (SD). The probability (P) values *p* ​< ​0.05 were considered statistically significant.

## 3 Results

### 3.1 Physicochemical characterization of MBGNPs

**Fig. 2:**
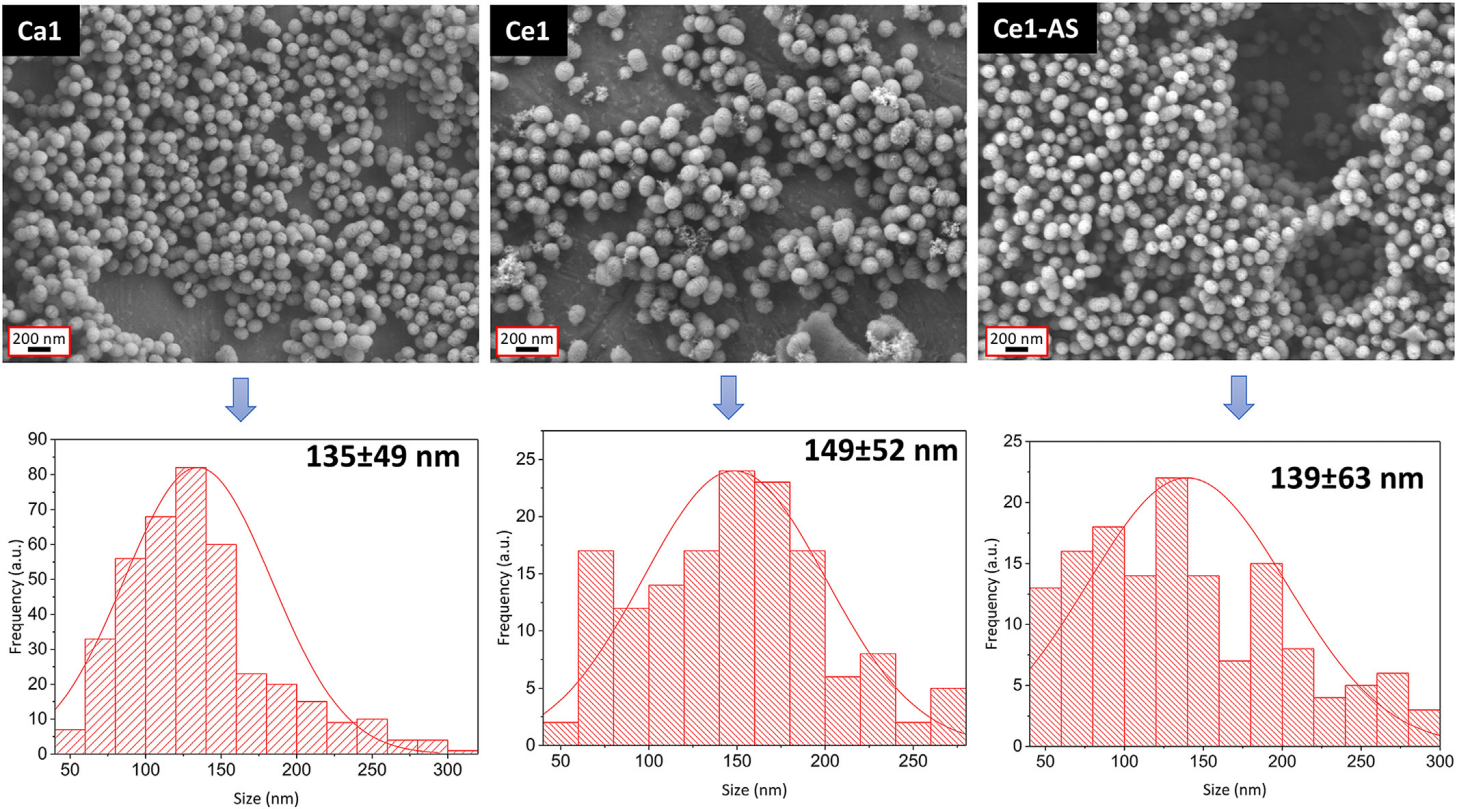
SEM images and particle size distribution of MBGNPs.

**Fig. 3:**
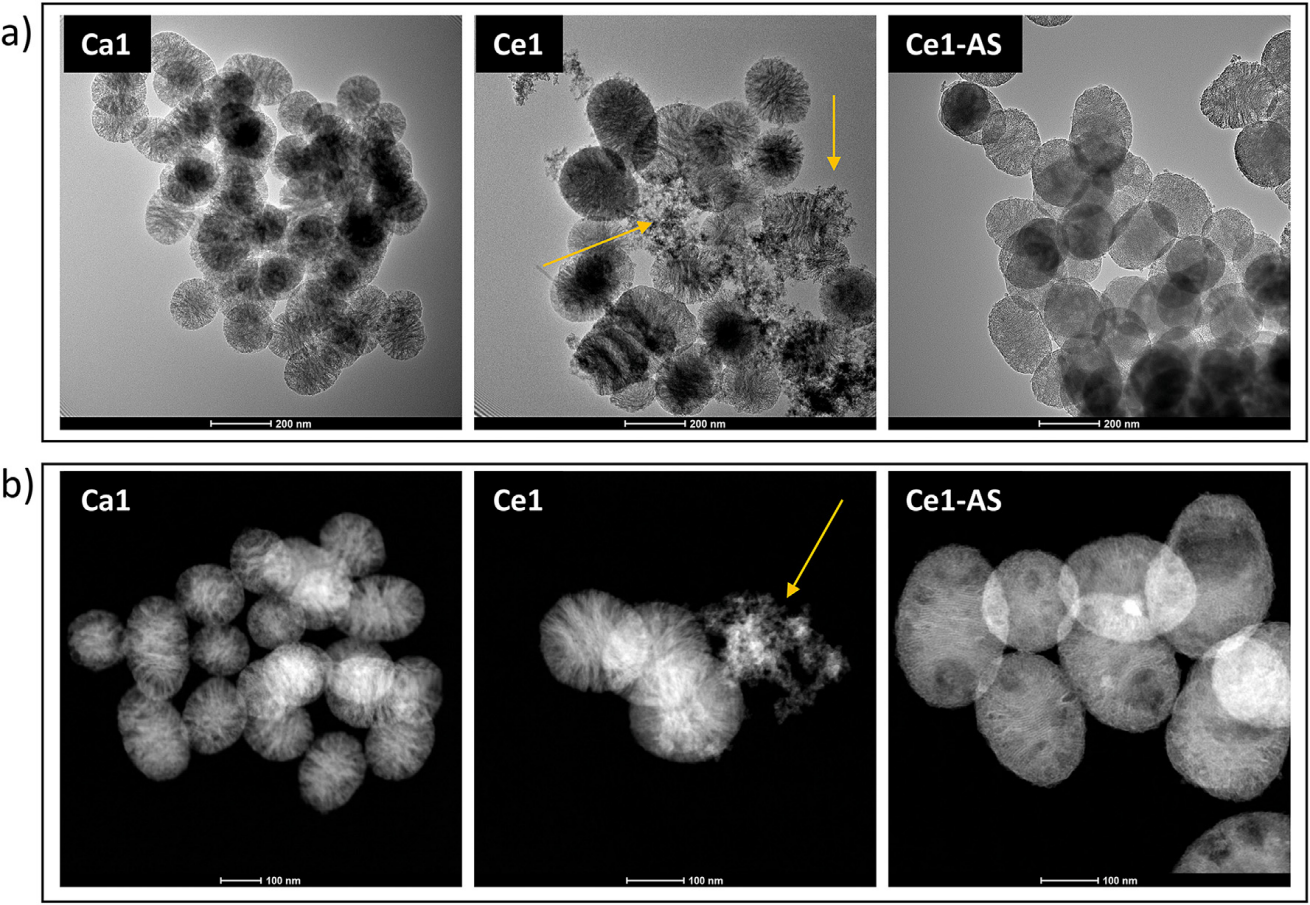
(a) TEM and (b) STEM images showing the morphology of MBGNPs (The arrows indicate nanoceria clusters).

The morphology of the synthesized mesoporous bioactive glass nanoparticles was examined by scanning electron microscopy (SEM) and (scanning) transmission electron microscopy ((S)TEM). SEM images (Fig. 2) show dispersed, spherical nanoparticles. As shown in Fig. 2, nanoparticles with an average size ranging between 100 and 200 ​nm were synthesized. The different synthesis methods of cerium doped nanoparticles did not significantly affect the size of the nanoparticles. The direct addition of cerium during the sol-gel synthesis resulted in the formation of nanoceria clusters in Ce1, as documented by the TEM and STEM images in Fig. 3 (indicated by arrows). Nanoceria was not observed in the samples prepared by the TIE method (Ce1-AS composition). The actual compositions of prepared MPBGNs (measured by ICP-OES) are shown in Table 1. The results show that the concentration of cerium is controlled by both synthesis methods, but in the Ce1-AS composition, the amount of calcium decreased significantly compared to the parent Ca1 glass.

**Table 1:** Actual compositions of MBGNPs (mol%) measured by ICP-OES.

MBGNPs Type	SiO_2_	CaO	Ce_2_O_3_
Ca1	90.3 ​± ​0.5	9.7 ​± ​0.5	–
Ce1	88.5 ​± ​0.2	10.1 ​± ​0.2	1.40 ​± ​0.03
Ce1-AS	91.7 ​± ​0.1	7.2 ​± ​0.1	1.1 ​± ​0.1

**Fig. 4:**
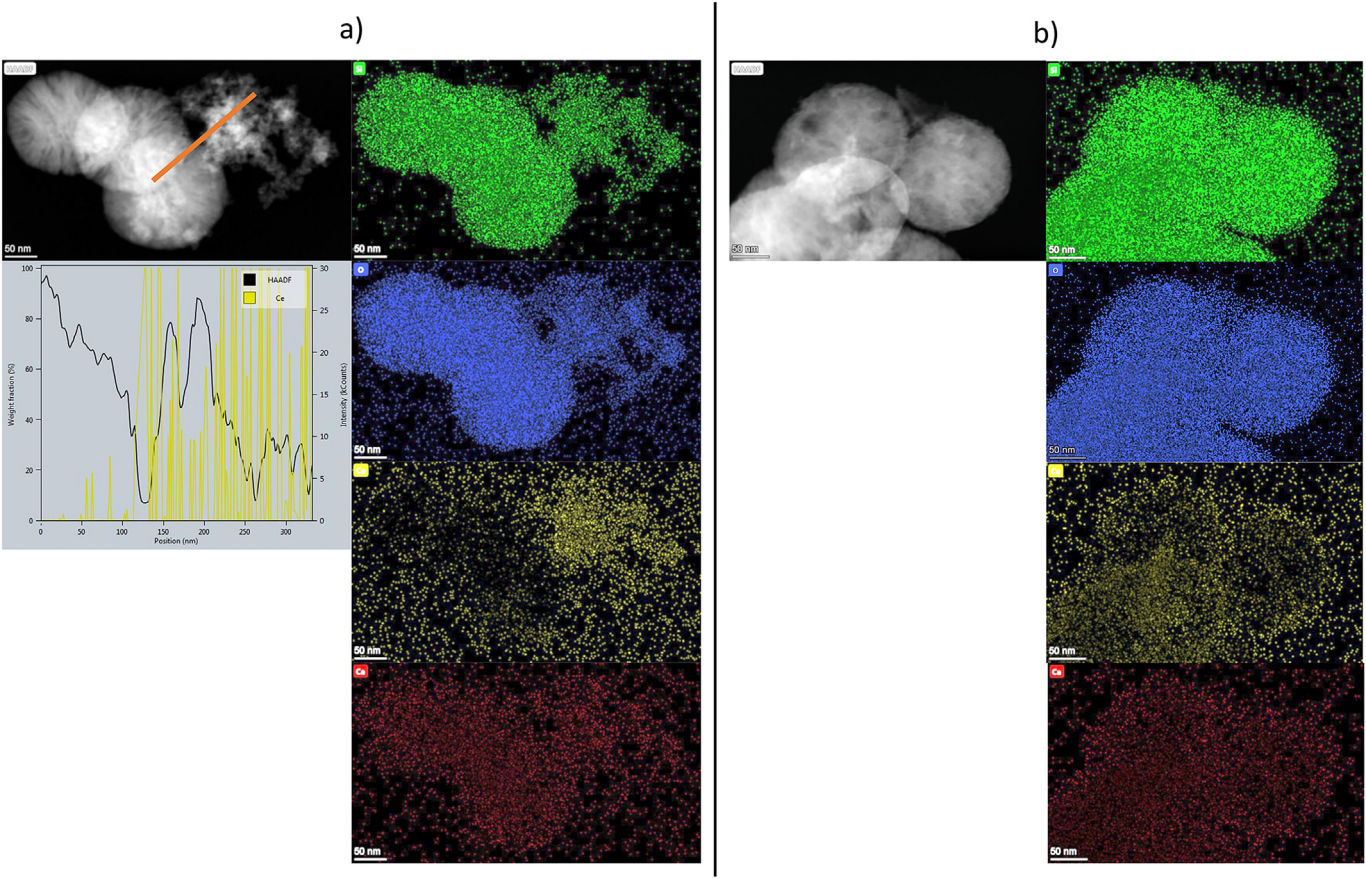
(a) EDX element mapping and line profile measurement of Ce1, and (b) EDX element mapping of Ce1-AS composition. The detected elements from top to bottom are Si (green), O (blue), Ce (yellow), and Ca (red).

Fig. 4 shows the EDX element mapping measurement of Ce1 and Ce1-AS compositions. For Ce1, cerium was mostly located in smaller clusters in the form of nanoceria. Due to the nature of the synthesis processes, which in both cases include calcination in an oxidation atmosphere at 650 ​°C, the presence of Ce exclusively in a 4+ oxidation state is expected. For observing possible differences between MBGNPs and side products, a line profile measurement was performed for Ce1. The line profile measurement of Ce1 confirmed that cerium is mostly located in these small clusters. In the Ce1-AS sample, cerium was distributed homogeneously and located on the surface of the glass nanoparticles.

**Fig. 5:**
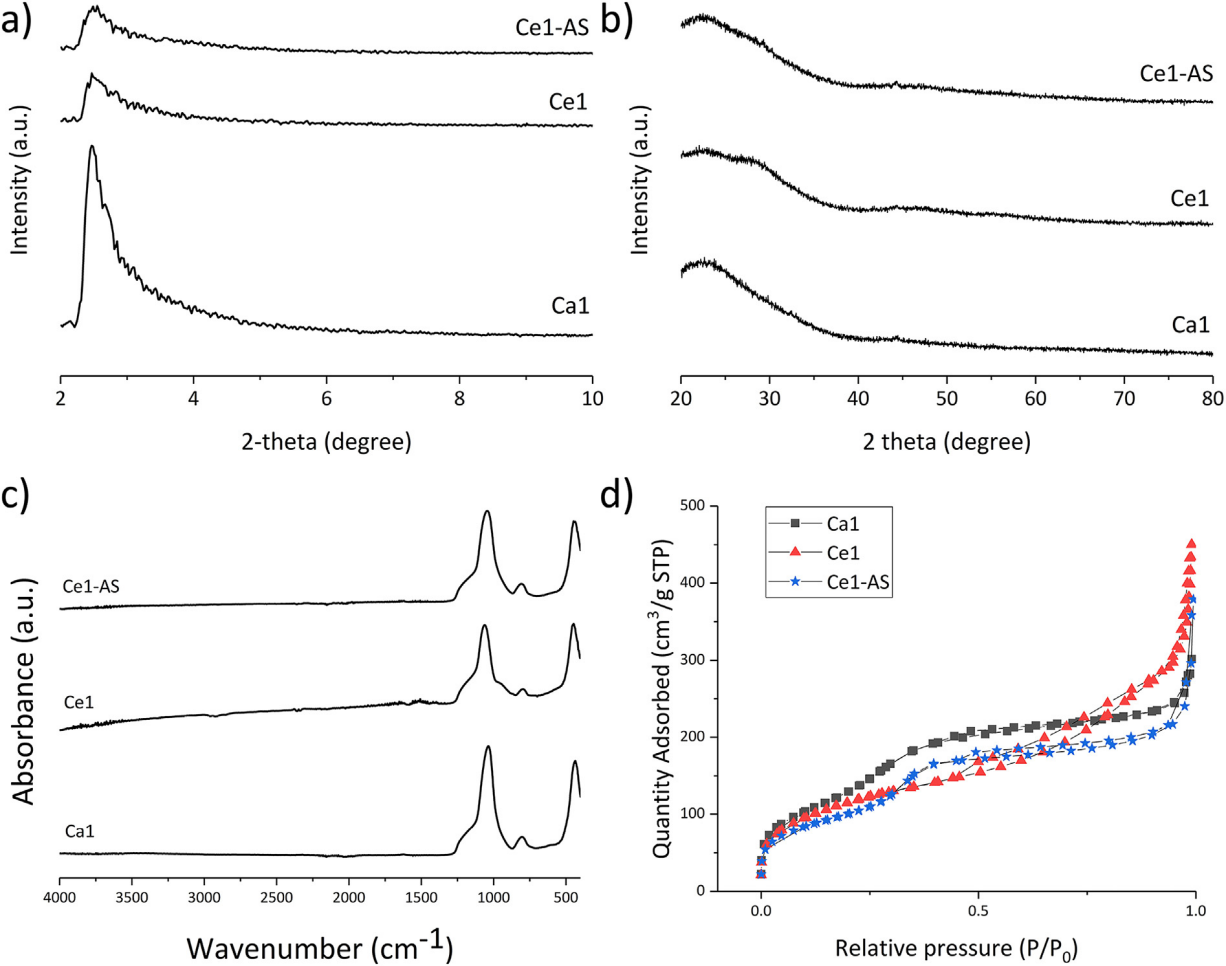
(a) and (b) X-ray diffraction patterns of Ca1, Ce1, and Ce1-AS showing the effects of the mesoporous structure between 2–10° and 20–80°; (c) Fourier-transform infrared spectroscopy (FTIR) spectra and (d) Nitrogen adsorption-desorption isotherm plots of Ca1, Ce1, and Ce1-AS nanoparticles.

Diffraction patterns measured in a low 2Θ range, 2–10° (Fig. 5(a)), document the mesoporous nature of the nanoparticles. XRD analysis performed in the 2Θ range 20–80° (Fig. 5(b)) did not show any discrete diffraction maxima, indicating the amorphous nature of the nanoparticles. The chemical structure of MBGNPs was studied by FTIR spectroscopy (Fig. 5(c)). All samples showed characteristic IR spectra corresponding to sol-gel-derived silicate glasses [39]. The band at 470 ​cm^−1^ was attributed to the Si–O–Si rocking mode [40]. The band at around 790 ​cm^−1^ was attributed to the Si–O–Si symmetric stretching vibration [39]. Moreover, the broad band located between 1000 and 1250 ​cm^−1^ was attributed to Si–O–Si stretching vibration and Si–O-(non-bridging oxygen) stretching vibration [39]. The synthesis method and the way of cerium addition had no impact on FTIR results.

N_2_ adsorption-desorption isotherms of the MBGNPs are shown in Fig. 5(d). Table 2 summarizes the specific surface area values, total pore volumes, and average pore diameters. H4-type hysteresis loops were observed for Ca1 and Ce1-AS. On the other hand, H3-type hysteresis loops were observed for Ce1 (Fig. 5(d)), indicating the presence of slit-shaped pores in Ce1 and narrow slit pores in Ca1 and Ce1-AS [41]. The addition of cerium decreased the specific surface area, especially with the TIE method, where the specific surface area decreased from 546 to 374 ​m^2^/g. On the other hand, the total pore volume did not change, but the mean pore size increased from 3.4 to 5.1 ​nm Ce1 has the highest pore volume and largest pore size of all prepared MBGNPs.

**Table 2:** Textural properties: S_BET_: Surface area, V_P_: Total pore volume, D_P_: Average pore diameter (Total pore volume at p/p0 ​= ​0.990).

MBGNPs Type	S_BET_ (m^2^/g)	V_P_ (cm^3^/g)	D_P_ (nm)
Ca1	546	0.46	3.4
Ce1	412	0.69	6.7
Ce1-AS	375	0.48	5.1

### 3.2 Ion release behavior of MBGNPs

**Fig. 6:**
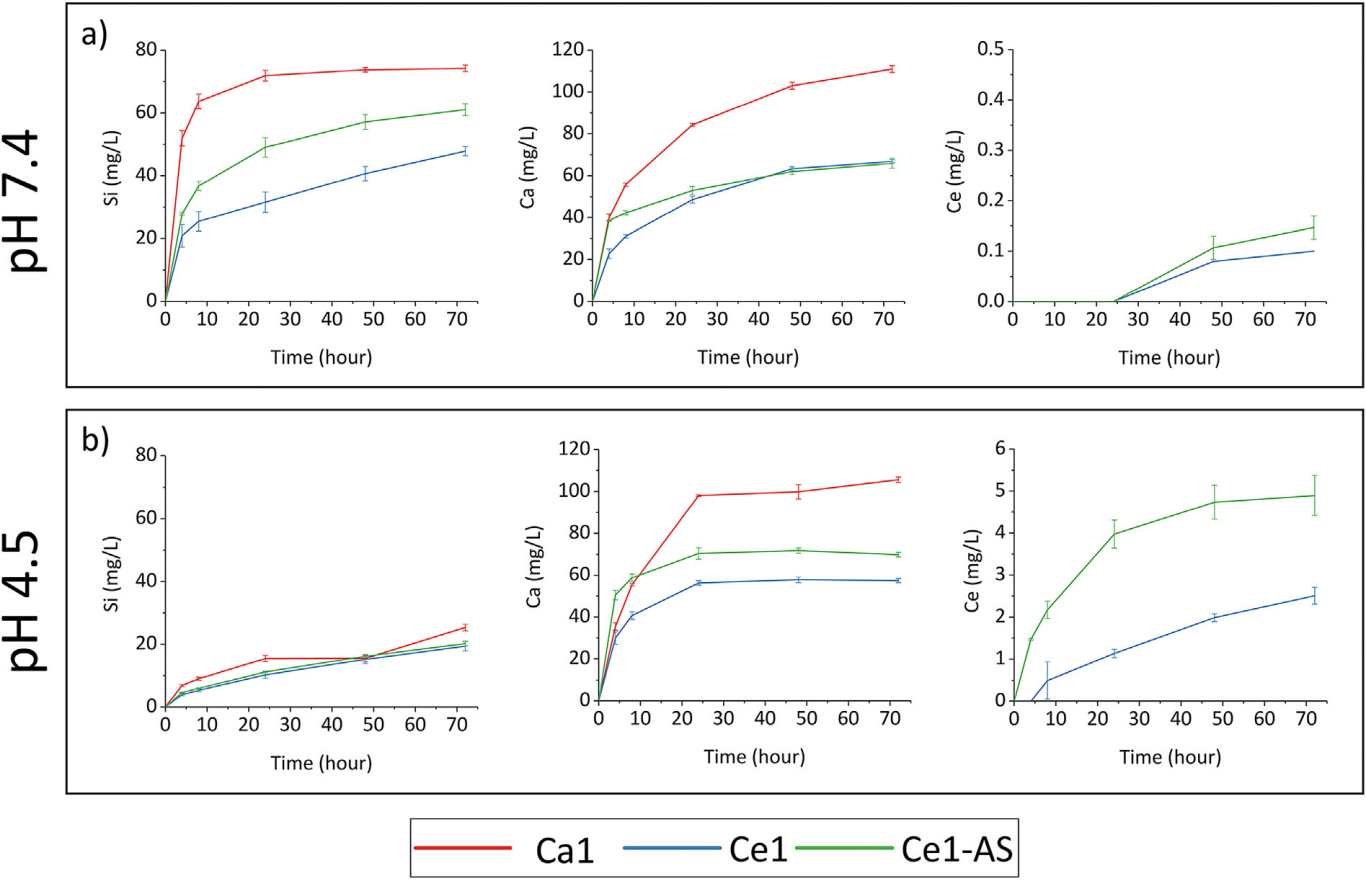
Ion release profiles of MBGNPs in (a) Tris/HCl buffer (pH 7.4) and (b) NaAC/HAC buffer (pH 4.5).

Ion release profiles of MBGNPs are shown in Fig. 6. It is essential to study the dissolution behavior of MBGNPs to understand the link between synthesis method, physicochemical properties, and their further biomedical applications. The ion release study was performed as a function of time and pH. Tris/HCl buffer (pH 7.4) and acetate buffer (pH 4.5) were used to study the pH influence on the ion release. The release of Si was faster at pH 7.4, while the acidic environment (pH 4.5) promoted the release of Ce. The addition of cerium slowed down the release of calcium at both tested pH values compared to undoped nanoparticles. Moreover, the change of pH did not affect the release of calcium, which was similar for both pH values.

### 3.3 Protein adsorption

**Fig. 7:**
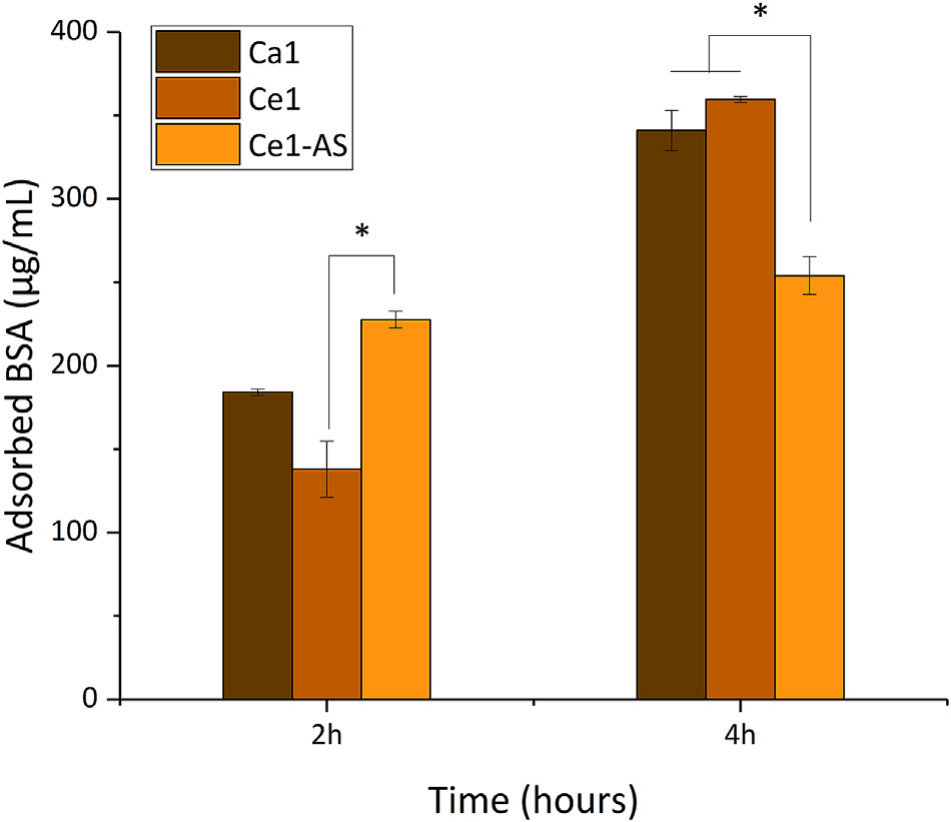
Adsorption of bovine serum albumin (BSA; μg/mL) on the surface of MBGNPs.

Fig. 7 shows the concentration of adsorbed BSA by MBGNPs. The amount of adsorbed BSA increased over time in all samples. Ca1 and Ce1 compositions did not show any significant difference. On the other hand, within the first 2 ​h, Ce1-AS composition adsorbed a higher amount of protein compared to Ca1 and Ce1. Then the adsorption capacity of Ce1-AS decreased, and it absorbed a lower amount of BSA after 4 ​h.

### 3.4 In-vitro biocompatibility

**Fig. 8:**
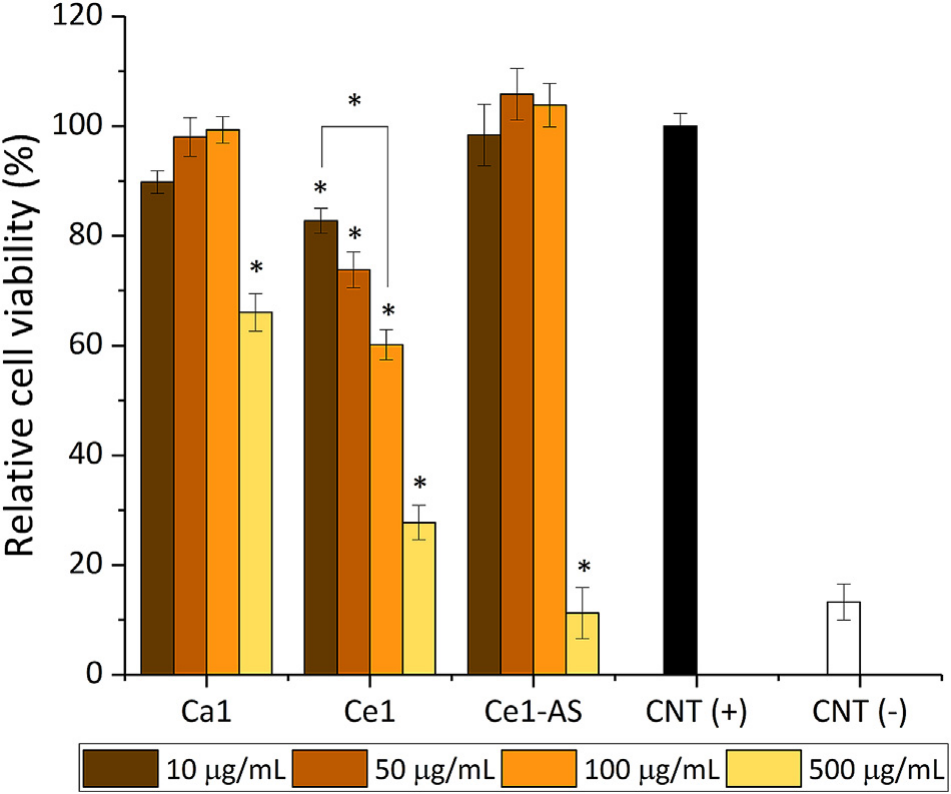
Relative viability of MC3T3-E1 cells cultured directly with MBGNPs (n ​= ​9, CNT = control, samples in triplicate, ∗*p* ​< ​0.05).

MC3T3-E1 cell viability in direct contact with the MBGNPs is shown in Fig. 8. The effect of the direct addition of the nanoparticles on the proliferation of MC3T3-E1 cells was studied by measuring the cell mitochondrial activity. The absorbance of positive control represented by the cells cultured only with cell culture medium was normalized to 100%. Compared to the control, direct addition of the Ca1 and Ce1-AS MBGNPs did not show any negative effect on the viability of MC3T3-E1 cells at concentrations of up to 100 ​μg/mL. On the other hand, Ce1 decreased the cell viability significantly with the addition of 10 ​μg/mL, and the cell viability continued to decrease by increasing the concentration of Ce. Lastly, with each type of MBGNPs, cell viability decreased at 500 ​μg/mL. Ce1-AS composition showed the most toxic effect on the cells: it showed similar cell viability values as the negative control.

**Fig. 9:**
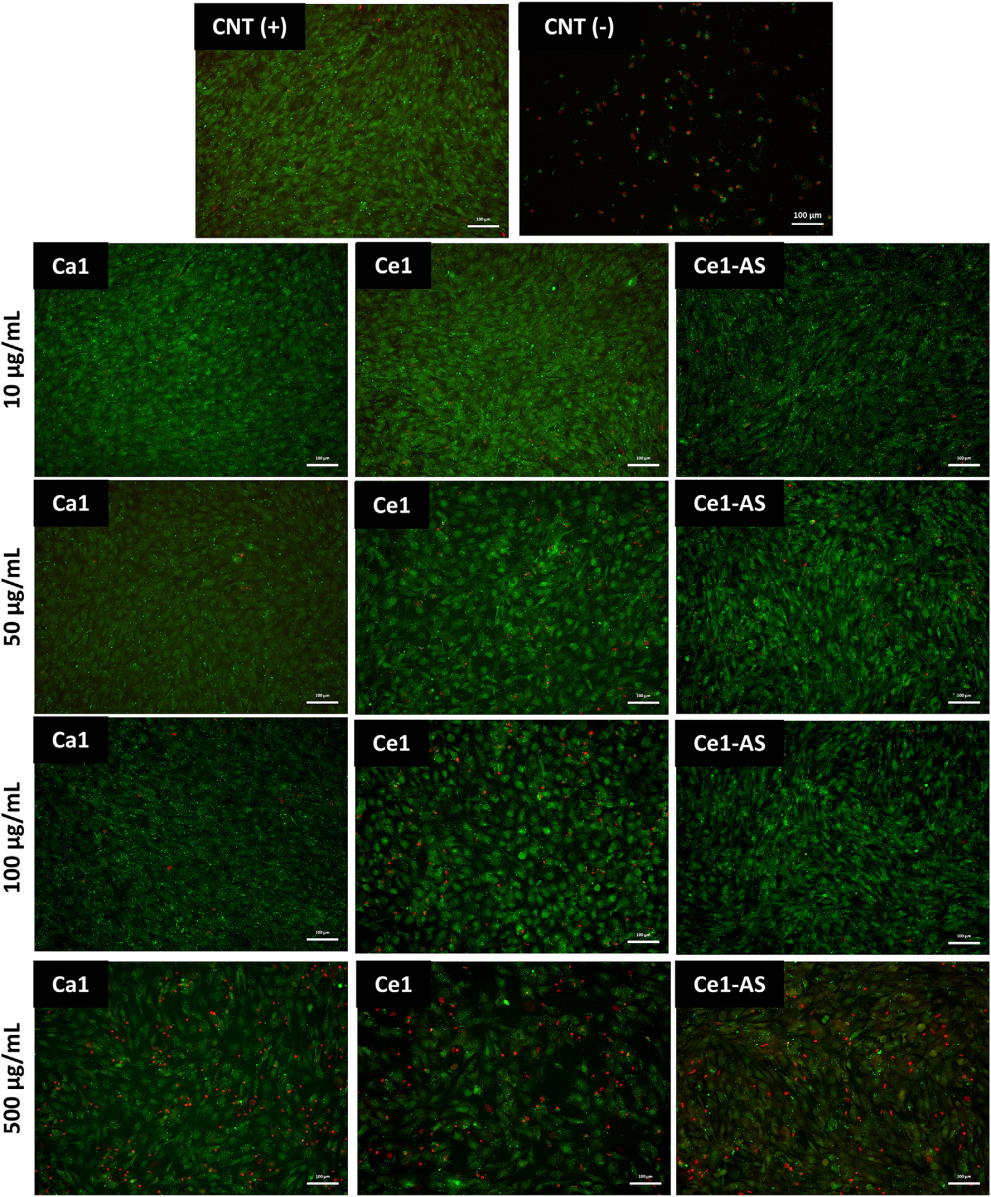
Live/dead staining images of Calcein-AM/PI stained MC3T3-E1 cells cultured directly with MBGNPs. CNT (+) images show MC3T3-E1 cell after 48 ​h in the absence of nanoparticles, and CNT (−) images show MC3T3-E1 cell after 48 ​h, cultured with 6 ​vol% DMSO containing cell culture medium (Scale bar ​= ​100 ​μm).

Fig. 9 shows the images of Calcein-AM/PI stained MC3T3-E1 cells cultured directly with MBGNPs. Calcein-AM (green) stains selectively live cells and PI (red) stains selectively dead cells. Staining with Calcein-AM/PI was carried out after 48 ​h to observe the effect of direct MBGNPs addition. The live/dead assay shows results in line with the cell viability study.

**Fig. 10:**
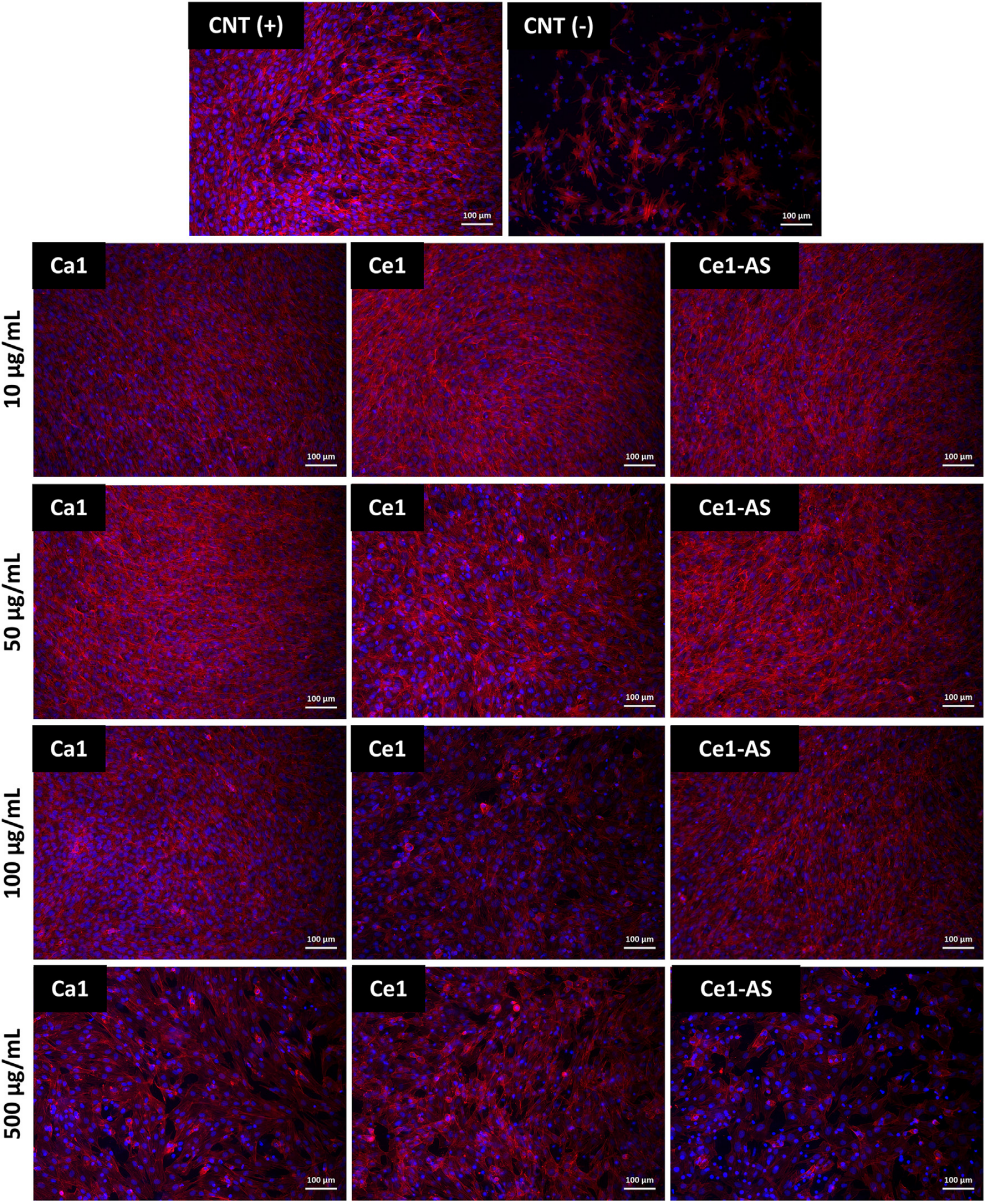
Fluorescence microscopy images of MC3T3-E1 cells after 48 ​h in direct contact with MBGNPs. CNT (+) images show MC3T3-E1 cell after 48 ​h in the absence of nanoparticles, and CNT (−) images show MC3T3-E1 cell after 48 ​h, with 6 ​vol% DMSO containing cell culture medium (scale bar ​= ​100 ​μm).

The morphology of MC3T3-E1 cells in the presence of a different concentration of MBGNPs was observed by fluorescence microscopy after staining with rhodamine phalloidin (for F-actin filaments in red) and DAPI (for nuclei in blue) (Fig. 10). The preosteoblast cells exhibit their phenotypical morphology, and they adhere to the surface of the well plate in the presence of MBGNPs. Up to 50 ​μg/mL nanoparticle concentration, all the samples showed higher cell density in comparison to the positive control. Additionally, the morphology and the spreading of the cells were similar for all samples up to the NP concentration of 100 ​μg/mL. Cell density decreased considerably with 500 ​μg/mL concentration according to fluorescence images.

### 3.5 In-vitro anti-inflammatory study

**Fig. 11:**
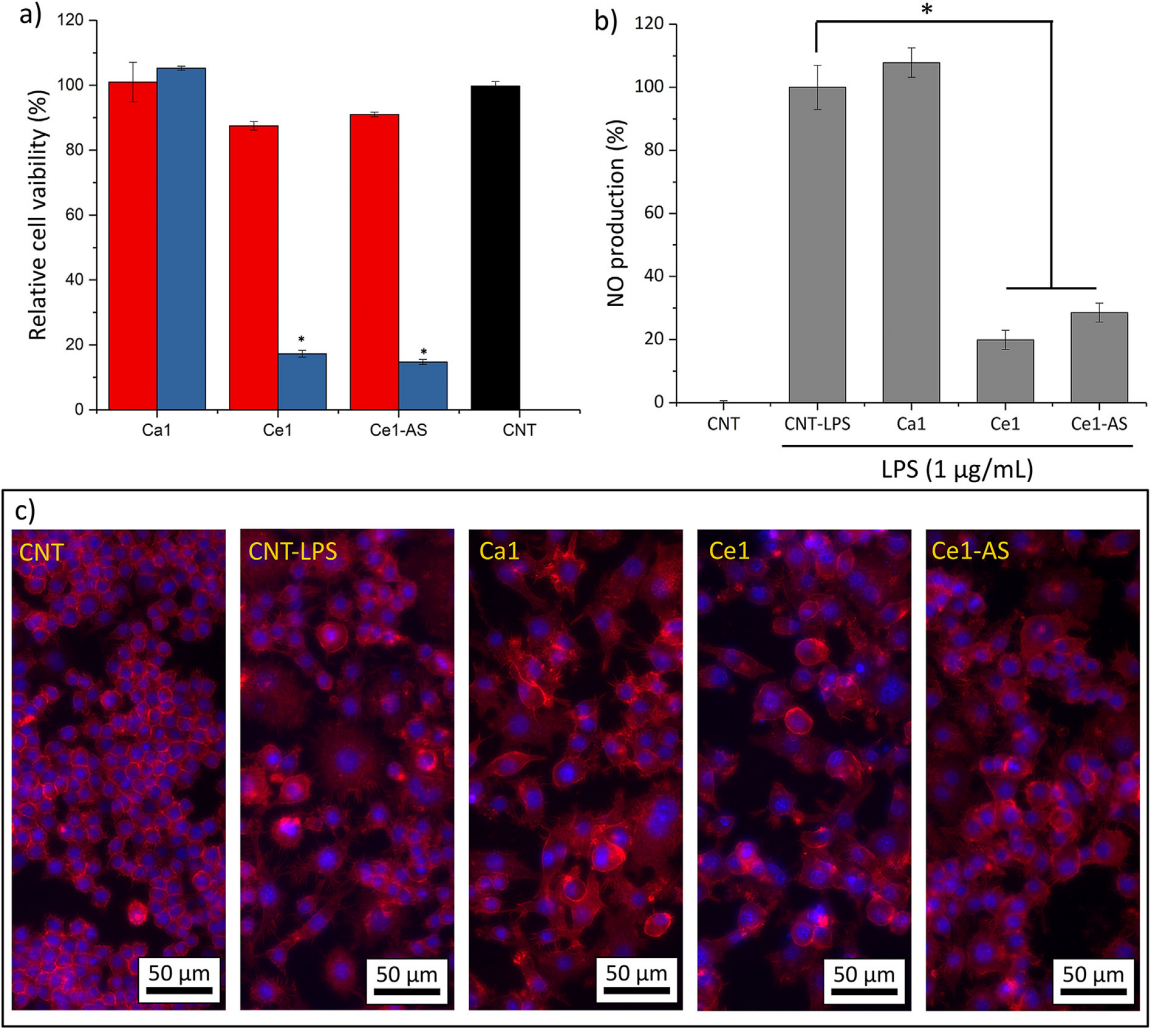
**a)** Relative viability of RAW 264.7 ​cells cultured directly with MBGNPs (n ​= ​9, CNT ​= ​control, samples in triplicate, ∗*p* ​< ​0.05). **b)** LPS-stimulated RAW 264.7 ​cells, analyzed by the production of nitric oxide (NO) (n ​= ​3, CNT ​= ​control, samples in triplicate, ∗*p* ​< ​0.05). **c)** Fluorescence microscopy images of LPS-stimulated RAW 264.7 ​cells after 24 ​h in direct contact with MBGNPs.

For determining the effective concentration of MBGNPs for anti-inflammatory study, a cytotoxicity test was performed with up to 100 ​μg/mL MBGNPs concentration. Fig. 11a shows relative cell viability after 48 ​h. The viability of RAW 264.7 ​cells was not affected by the presence of 50 ​μg/mL MBGNPs, irrespective of the MPBGNs composition. This concentration was therefore selected for further study. For evaluating the anti-inflammatory effect of MBGNPs, the production of NO was measured (Fig. 11b). LPS-induced cells produced significantly more NO than the non-induced cells. The treatment with cerium-containing MBGNPs significantly reduced the production of NO, where MBGNPs without cerium did not affect the production of NO. It is evident that cerium-containing MBGNPs drive cells to the anti-inflammatory stage. For visualizing the changes in the morphology of macrophages, the cells were stained with phalloidin and DAPI (Fig. 11c). Control group cells, which are without treatment, were found to be small and rounded. LPS-induced cells, on the other hand, were irregularly shaped and bigger in size. The treatment with Ca1 did not affect the morphology of LPS-induced macrophage cells. However, Ce1 and Ce1-AS treated cells were larger, and some of them showed flattened phenotypes.

### 3.6 Antibacterial activity

**Fig. 12:**
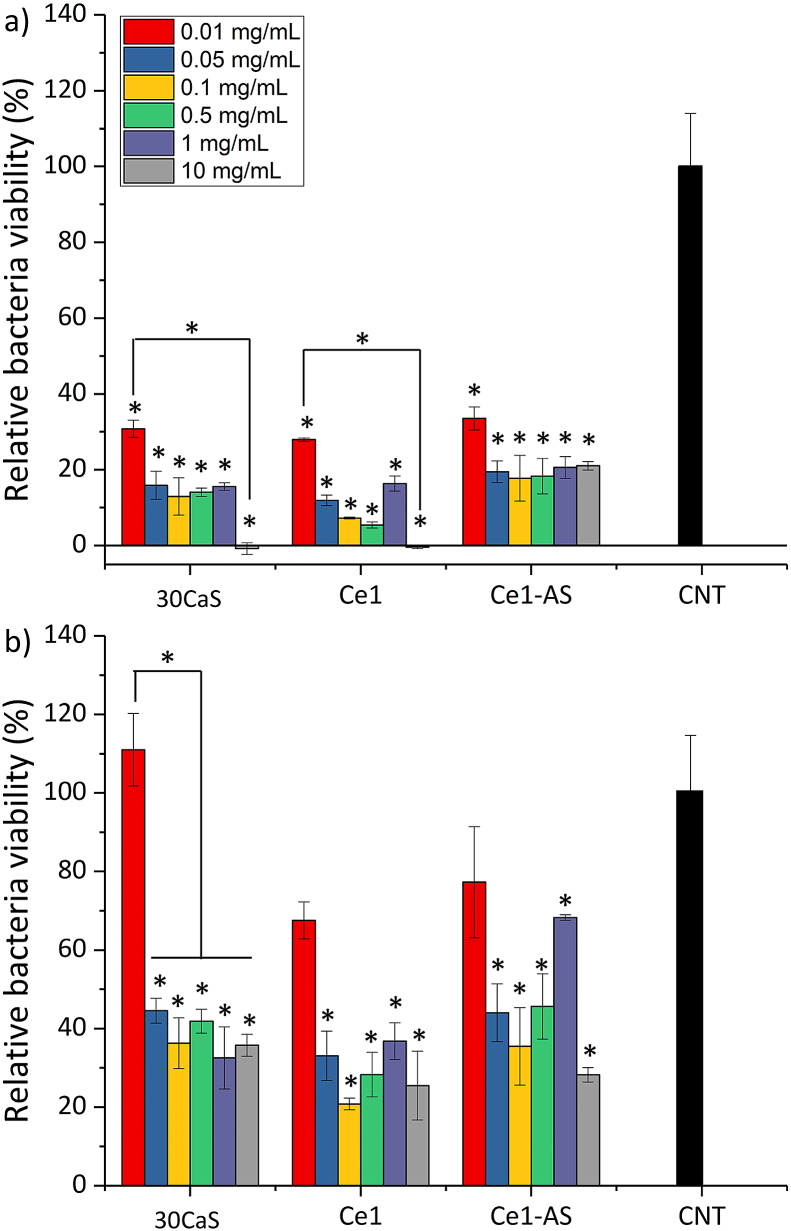
Antibacterial activity of MBGNPs after 24 ​h incubation with **a)***S. aureus* (Gram-positive) and **b)***E. coli* (Gram-negative) bacteria (n ​= ​3, samples in triplicate, ∗*p* ​< ​0.05).

*S. aureus* (Gram-positive) and *E. coli* (Gram-negative) bacteria were used in order to evaluate the antibacterial potential of the MBGNPs. Fig. 12 shows the relative bacteria viability after 24 ​h of contact with up to 10 ​mg/mL of MBGNPs. All compositions of MBGNPs showed antibacterial activity against both bacteria strains. Except for the 0.01 ​mg/mL concentration for gram-negative bacteria, all the samples significantly reduced the viability of both bacteria strains. Ca1 and Ce1 compositions showed a similar effect to the gram-positive bacteria. According to the measured OD values, Ca1 and Ce1 have completely inhibited the gram-positive bacteria growth with 10 ​mg/mL concentration. The Ce1-AS composition also reduced bacteria growth. On the other hand, the concentration of Ce1-AS did not significantly affect the gram-positive bacteria viability. Gram-negative bacteria were not affected at the nanoparticle concentration of 0.01 ​mg/mL, and the bacteria viability decreased by increasing the concentration of MBGNPs. Compared to the gram-positive bacteria, gram-negative bacteria were affected less by the presence of MBGNPs.

## 4 Discussion

Mesoporous bioactive glass nanoparticles were synthesized by a microemulsion-assisted sol-gel method. This technique allows a dynamic assembly process for obtaining MBGNPs. The system is formed by CTAB, EA, ammonium hydroxide, and water. The cationic surfactant CTAB was used as a templating agent to form a mesoporous structure. Microemulsion droplets acted as a barrier and prevented the nanoparticles from aggregation [10]. The microemulsion is a dynamic system, and microemulsion droplets frequently collide with each other [42], ensuring homogeneity of the chemical composition of the nanoparticles. On the other hand, the nanoparticles may vary in size due to this dynamic environment. Biologically active metal ions are commonly added to bioactive materials in order to improve their biological behavior [10], but the addition of metallic ions to mesoporous nanoparticles has challenges. Metallic ions could interact with the cationic surfactant (CTAB) and disrupt the mesoporous structure. They could also affect the silica nanoparticle’s surface charges, and as a result, agglomeration of the particles can occur [10]. Metal ions can be added either during the hydrolysis and condensation of TEOS or after the formation of silica nanoparticles. In this work, Ce^3+^ was added to nanoparticles utilizing both approaches. In the first approach, Ce^3+^ was added to the glass system directly during the synthesis, while in the second approach, Ce^3+^ was added to the as-synthesized MBGNPs via the postmodification method (Fig. 1).

Moreover, there is a difference between the nominal compositions of the MBGNPs and their actual compositions measured via ICP-OES (Table 1). A low ​amount of calcium was incorporated into the nanoparticles compared to the nominal compositions. This is a common issue with bioactive glass nanoparticles obtained via sol-gel chemistry [[43], [44], [45]]. This difference is mainly attributed to the washing steps before drying the nanoparticles [43]. Some of the precursors of calcium can be removed from the system during the centrifugation and washing steps of the MBGNPs after synthesis.

SEM images (Fig. 2) confirmed that the prepared nanoparticles were spherical in shape, well dispersed, and without agglomeration. The sizes of all MBGNPs were comparable (Fig. 2). Direct addition of cerium resulted in a similar shape, surface morphology, and particle size (Fig. 3). On the other hand, the direct addition of cerium also resulted in the formation of nanoceria as a side product, as documented by the TEM images of the Ce1 composition. In this case, cerium appears to be concentrated on the surface of the synthesized MBGNPs particles in the form of nanoceria clusters. In the Ce-AS particles, ceria is distributed homogeneously on the surface of synthesized nanoparticles. This result was confirmed with EDX line profile measurements. In Fig. 4a, the presence of cerium increased as the arrow moved from its starting position toward ​the cluster of smaller nanoparticles. Nanoceria could be formed under basic conditions, which were also used in the synthesis of MBGNPs in this work [46]. Nanoceria can interact with several reactive oxygen species (ROS) and intermediates (ROI), so it has potential uses in a wide range of biological applications [23,46]. For example, Pinna et al. [23] deliberately synthesized nanoceria in-situ on the surface of mesoporous silica nanoparticles (MSNs) for their antioxidant and osteogenic properties. However, nanoceria was not detected by XRD in this study ​and was only observed in high magnification TEM micrographs. The absence of a diffraction pattern was attributed to the low amount of nanoceria, which was below the detection limit of XRD. Goh et al. [33] used a sol-gel approach to prepare cerium-containing bioactive glass nanoparticles (BGNs), and the presence of nanoceria was detected by XRD analysis for cerium concentrations higher than 1 ​mol%.

In the present study, the goal was the incorporation of Ce in the silicate structure of the MBGNPs, and not to form nanoscale ceria. Thus, to avoid the formation of nanoceria, cerium was incorporated into MBGNPs through a postmodification method called template ion exchange (TIE). TIE and similar strategies have been widely used for the addition of metal ions to silica nanoparticles [20,37,47,48]. Cerium was successfully incorporated into MBGNPs without the formation of nanoceria, as evidenced by TEM images (Fig. 3). The Ce1-AS composition showed similar particle size dispersity in comparison to the cerium-free counterpart, but the surface of the nanoparticles was modified, as documented by the TEM micrographs (Fig. 2). After postmodification, the surface of Ce1-AS nanoparticles was smoother than that of Ca1. The nanoscale topography has a decisive influence on the interactions of nanoparticles with proteins and cells, thus having the possibility to control the surface topography of the particles is important [49].

Irrespective of the synthesis method used, the addition of cerium disrupted the ordered pore structure of MBGNPs, as confirmed by the low angle XRD measurements (Fig. 5a). Ordered mesoporous structured silica nanoparticles exhibit distinct diffraction patterns at low 2θ angles (2–10°) [50]. For instance, one of the most studied materials, silica MCM-41, shows a hexagonal symmetry of the pore ordering, and it typically contains four main reflection lines or more at low angles [51]. The low-angle diffraction patterns shown in Fig. 5a contain only one broad resolved diffraction peak, with relatively low intensity, indicating deteriorated and poorly ordered pore structure [52]. The ordered mesoporous structure was therefore not confirmed in the mesoporous silica systems with added calcium. For Ce1 and Ce1-AS compositions, the addition of cerium further decreased the intensity of the diffraction peak at ​​2≥ 2​θ 2.7°, indicating an increased disruption of the ordered mesoporous structure.

Nitrogen adsorption analysis provides complementary data to the results of TEM and XRD analyses on the mesoporous structure of synthesized nanoparticles. Ca1 and Ce1-AS compositions showed similar adsorption/desorption curves (Fig. 5d) with H4-type hysteresis loops. However, the pore sizes in Ce1-AS increased when the TIE method was applied. As a result, the specific surface area of Ce1-AS decreased to 375 ​m^2^/g (Table 2). Cerium addition with the TIE method resulted in reduced pore volume ​and probably clogged pore structure. The Ce1 composition showed H3-type hysteresis loops, which indicates narrow slit pores compared to the Ca1 composition [41], and also the largest average pore size of 6.7 ​nm (Table 2). In literature, average pore sizes of around 3 ​nm have been reported when CTAB was used as a pore templating agent in MSNs [53]. The larger pore size documented in the present study was attributed to the interaction of additional calcium and cerium with CTAB. The molecular structure of MBGNPs was studied by FTIR (Fig. 5(c)). No absorption bands attributed to C–H stretching (3000–2840 ​cm^−1^) vibrations were visible in the FTIR spectrum [54], confirming complete removal of the CTAB during calcination.

We further investigated the dissolution mechanism of Ca1, Ce1, and Ce1-AS as a function of time and pH. Acetate buffer (pH 4.5) and tris buffer (pH 7.4) were used to study the pH influence on the degradation of nanoparticles (Fig. 6). Buffer solutions containing phosphate such as simulated body fluid (SBF), phosphate buffer saline (PBS), and cell culture medium, are not suitable for investigating the release of cerium and studying the effect of pH ​and because cerium can form insoluble complexes with phosphate, and the amount of released cerium cannot be determined by ICP-OES [20,55], the pH of these media is not stable and changes with time [56]. The release of cerium was more rapid at pH 4.5, and the release of silica was faster at pH 7.4. This result indicates that cerium was preferentially released at lower pH conditions. This property of Ce containing MBGNPs can be advantageous in biomedical applications as it may increase the bactericidal properties of the nanoparticles in severe bacterial infections [57]. Moreover, cerium has shown some anti-cancer activity: the release of cerium will be faster in a tumor environment with lower pH ​but slower in the bloodstream [58]. Additionally, Ce release from Ce1-AS composition was significantly higher than from Ce1. This result could be explained by the different synthesis methods of Ce1 and Ce1-AS. With the TIE method, cerium was incorporated mainly on the surface of the nanoparticles. On the other hand, cerium could be distributed evenly in the Ce1 nanoparticle structure since the cerium precursor was added during the hydrolysis and condensation of TEOS; however, TEM results (Fig. 4a) showed that most of the cerium concentrated in the nanoceria clusters, not in the Ce1 MBGNPs.

Biomaterial-protein interactions are of key importance as they determine the biological properties of solid materials. When biomaterials are in contact with body fluids, they get surrounded by proteins. In a short time, proteins are adsorbed on the surface of biomaterials, and the subsequent biological interactions with the materials depend on the characteristics of the protein attachment [59,60]. Numerous parameters affect the interaction between a biomaterial and proteins, including surface chemical composition, biomaterial characteristics (particle size, surface charge, hydrophobicity, crystallinity, and surface topography), and protein properties (dimension and molecular weight) [59]. In this study, all MBGNPs showed the ability to adsorb BSA, which was selected as a model protein (Fig. 7). Ca1 and Ce1 adsorbed a similar amount of BSA, without significant difference. On the other hand, Ce1-AS showed faster adsorption during the first 2 ​h, and Ce1-AS exhibited significantly higher protein adsorption than Ce1. However, Ce1-AS stopped adsorbing protein ​at the same point, while Ca1 and Ce1 continued to adsorb an increasing concentration of proteins over time. This behavior could be explained by the different topography at the nanoscale of the nanoparticles. Ca1 and Ce1 compositions showed a rough surface and higher specific surface area (Table 2) compared to Ce1-AS. Ca1 and Ce1 exhibited rougher surfaces, which could make the initial adsorption more difficult, yet the adsorption could proceed for a longer period of time as the protein-specific surface areas of these nanoparticles are larger. Another reason for the different protein adsorption characteristics could be related to the composition of the glasses. Recently, Kapp et al. [60] conducted a study on the protein adsorption capacity of SiO_2_–CaO bioactive glass nanoparticles. The experimental results indicated that the adsorption of protein changed regardless of the particle composition up to 2 ​h. However, the glasses with higher Ca concentrations showed a higher amount of protein adsorption in further incubations. These findings are in agreement with this study, and the actual composition of Ce-AS (Table 1) demonstrated a reduction in Ca concentration.

Bioactive glass compositions and concentrations are the key parameters determining their possible cytotoxicity [10]. In general, nanoparticles can cause cytotoxicity for numerous reasons [61]. Zheng et al. [20] studied the cytotoxicity of up to 0.2 ​mol% cerium-containing MBGNPs toward ​fibroblast cells. The study showed that cerium-containing MBGNPs with 1 ​mg/mL concentration (without direct contact with the cells) did not show any cytotoxic behavior toward ​fibroblast cells. Moreover, Atkinson et al. [28] studied the cytotoxicity of up to 5 ​mol% of cerium-containing MBGs. Likewise, the MBGs did not show cytotoxic behavior toward ​fibroblast cells during the indirect test. In the present study, the cytotoxicity of MBGNPs toward ​MC3T3-E1 preosteoblast cells was assessed using the WST-8 assay, which measures cellular activity (Fig. 8a). In this study, the cytotoxicity was evaluated by the direct addition of the MBGNPs in cell culture medium in the amount of up to 500 ​μg/mL. Ca1 and Ce1-AS showed non-cytotoxic behavior toward ​MC3T3-E1 cells up to 100 ​μg/mL. On the other hand, Ce1 reduced cell viability significantly. Ce1-AS released a higher amount of cerium at physiological pH (7.4, Fig. 6) compared to Ce1, but only Ce1 affected cell viability at lower amounts of nanoparticles. This result can be explained by the presence of nanoceria, which is a by-product of the Ce1 synthesis, as mentioned above. The reason for toxicity could be related to the physicochemical properties since nanoceria shows DNA damaging potential and cell death, which has been reported to correlate with induced oxidative stress production compared to the micron-sized counterparts [62]. Significant cell loss has been observed in a dose-dependent manner of nanoceria toward ​human neuroblastoma cells [62]. In the present study, all prepared MBGNPs showed cytotoxic behavior with 500 ​μg/mL, especially Ce1 and Ce1-AS. At such a high concentration, the amount of released cerium ions is likely to be above the therapeutic level (Fig. 8). The results of the live/dead assay provided comparable results with the WST-8 assay (Fig. 9). The number of dead cells decreased by 100 ​μg/mL MBGNPs concentration for both Ca1 and Ce1-AS. Rhodamine phalloidin and DAPI staining showed MC3T3-E1 cells exhibiting their phenotypical morphology and adhering to the surface of the well-plate in the direct presence of MBGNPs.

Macrophage cells respond to the environment and polarize to two distinct phenotypes, namely M1 and M2 [63]. M1 are proinflammatory cells, which have a pathogen-killing ability. On the other hand, M2 macrophages are anti-inflammatory cells, which promote cell proliferation and tissue repair [63]. In this study, we treated macrophage cells with lipopolysaccharide (LPS) to cause inflammation and polarized the macrophages to the M1 phenotype [63,64]. LPS is an outer membrane component of Gram-negative bacteria, and it can cause inflammation. M1 proinflammatory cells produce nitric oxide (NO) [65], which is very critical in proinflammatory responses, and ROS may trigger the generation of NO [66]. Fig. 10b shows that macrophages stimulated with 1 ​μg/mL LPS released NO while the control group showed no release of NO. Both cerium-containing Ce1 and Ce1-AS compositions decreased the release of NO significantly, while Ca1 composition did not affect the NO release. NO assay indicated that cerium-containing MBGNPs inhibited proinflammatory cells and induced an anti-inflammatory response. This effect could be attributed to the redox activity of cerium. Cerium could change its oxidation state (Ce^4+^ and Ce^3+^), which gives cerium the ability to scavenge ROS and tune the oxygen in the microenvironment through changing the oxidation state [20,23]. Therefore, cerium could promote an anti-inflammatory response [20,23]. The fluorescence images of the macrophages support the results of NO measurements (Fig. 10c). Without LPS addition, the macrophages were found to be small and rounded in shape. The LPS induced cells were larger in size and had an irregular shape. Moreover, some of the cells elongated into a spindle-shaped morphology, and their appearance fitted with the M1 macrophage (proinflammatory) phenotype [64]. On the other hand, the cells treated with Ce containing MBGNPs showed flattened, enlarged shape, being bigger than the cells in the control group, which were similar to M2 macrophages (anti-inflammatory) [64]. Further studies such as quantification of multiple cell surface markers and cytokine profiles will be helpful to determine the role of Ce containing MBGNPs in macrophage polarization.

One of the biggest challenges for the healthcare system is bacterial contamination of implants. The use of implants has increased with the increase of the aging population, with the direct increase in the risk of bacterial contamination [67]. Moreover, many strains of bacteria develop resistance to antibiotics, making the healing process more difficult [12]. There is currently a pressing need for the development of antibiotic-free antibacterial materials, and in this context, bioactive glasses have great potential to deliver therapeutic ions with antibacterial properties such as cerium [12]. Cerium shows antibacterial activity against Gram-positive and Gram-negative bacteria through ROS production. In a physiological pH environment, cerium scavenges ROS, but in a lower pH environment caused by bacterial infection, cerium generates ROS and damages bacteria [25,68]. *S. aureus* (Gram-positive) and *E. coli* (Gram-negative) bacteria were used to determine the antibacterial activity of MBGNPs (Fig. 12). We used a higher amount of MBGNPs (up to 10 ​mg/mL) than in cell studies to determine the minimum inhibitory concentration. Except for the 0.01 ​mg/mL concentration for gram-negative bacteria, all compositions of MBGNPs showed antibacterial activity against both bacteria strains. MBGNPs significantly reduced the relative bacteria viability. Ca1 and Ce1 compositions showed a similar effect to Gram-positive bacteria, and with 10 ​mg/mL they inhibited Gram-positive bacteria growth completely. On the other hand, the Ce1 and Ce1-AS effect did not change significantly with the increased concentration of nanoparticles. This result could be explained by the bioactive glass role in pH change. It is known that bioactive glasses increase the pH locally when they dissolve [56]. The release of cerium (Fig. 6) and its antibacterial ability decreased with increasing pH. Gram-negative bacteria were less affected by nanoparticles than gram-positive bacteria. This different action of MBGNPs against the two bacteria strains could be explained by differences in their cell walls: the Gram-positive bacterial cell walls contain a thick peptidoglycan layer, which is attached to teichoic acids, influencing the interaction of bacteria cells with Ce [69].

Summarizing, different routes of cerium addition affect the nanomorphology and ion release properties of cerium-containing MBGNPs. Moreover, this study evidenced the biocompatibility of cerium-containing MBGNPs and their anti-inflammatory and antibacterial activities.

## 5 Conclusion

Cerium-containing MBGNPs were successfully synthesized using two different approaches, the microemulsion-assisted sol-gel method and the combination of the microemulsion-assisted sol-gel method and the template ion-exchange method (TIE). Well-dispersed spherical particles with sizes in the range of 100–200 ​nm were prepared. The addition of metallic ions did not affect the amorphous nature of the MBGNPs. TEM examination revealed the presence of nanoceria clusters in Ce1 nanoparticles prepared by the microemulsion-assisted sol-gel method. The TIE method allowed the preparation of nanoparticles (Ce1-AS) with a change in surface topography. The formation of nanoceria clusters was avoided with the TIE method, which yielded homogeneous nanoparticles and uniform distribution of cerium on their surfaces. Moreover, the direct addition of Ce1 nanoparticles to the cell culture medium showed higher cytotoxicity toward ​MC3T3-E1 preosteoblast cells up to 100 ​μg/mL, compared to Ce1-AS nanoparticles. These findings suggest that the TIE method is more favorable in terms of the synthesis of nanoparticles with desired properties. Both cerium-containing MBGNPs exhibited anti-inflammatory responses in culture with LPS-induced RAW 264.7 macrophage cells. Additionally, all samples showed antibacterial properties against *S. aureus* and *E. coli*. Because of their biocompatibility, anti-inflammatory, and antibacterial properties, the synthesized cerium doped MBGNPs show potential for various multifaceted biomedical applications, for example, to combat inflammatory bone diseases and bone infections.

## Credit author statement

**Fatih Kurtuldu**: Conceptualization, Methodology, Validation, Investigation, Data curation, Visualization, Writing - Original Draft. **Hana Kaňková:** Validation, Investigation, Writing – Review & Editing. **Ana M. Beltrán:** Validation, Investigation, Writing – Review & Editing. **Liliana Liverani:** Validation, Writing – Review & Editing. **Du**š**an Galusek:** Conceptualization, Writing – Review & Editing, Supervision, Project administration, Funding acquisition. **Aldo R. Boccaccini:** Conceptualization, Writing – Review & Editing, Supervision, Project administration, Funding acquisition.

## Declaration of competing interest

The authors declare that they have no known competing financial interests or personal relationships that could have appeared to influence the work reported in this paper.
